# Improved Periorbital Satisfaction After Combined Mid and Upper Facial Treatment With Hyaluronic Acid Fillers and OnabotulinumtoxinA: Effectiveness and Safety Results From a Post‐Marketing Study

**DOI:** 10.1111/jocd.70487

**Published:** 2025-10-21

**Authors:** Gregory J. Goodman, Pierre Cuvelier, Frank Lin, Cara McDonald, Sarah Boxley, Joan Vandeputte, Ivar Van Heijningen, Samira Baharlou, Traci Baker, Smita Chawla, Carola de la Guardia

**Affiliations:** ^1^ Department of General Practice Monash University Clayton Australia; ^2^ University College of London London UK; ^3^ Centre de La Fontaine Loverval Belgium; ^4^ Monash University Melbourne Australia; ^5^ St Vincent's Hospital Melbourne Australia; ^6^ SkinBox Clinics Perth Australia; ^7^ Private Practice Oudenaarde Belgium; ^8^ Duinbergen Clinic Knokke‐Heist Belgium; ^9^ Skin Immunology & Immune Tolerance (SKIN) Research Group Vrije Universiteit Brussel (VUB) Brussels Belgium; ^10^ Department of Dermatology Vrije Universiteit Brussel (VUB), Universitair Ziekenhuis Brussel (UZ Brussel) Brussels Belgium; ^11^ Allergan Aesthetics, an AbbVie Company North Chicago Illinois USA; ^12^ Allergan Aesthetics, an AbbVie Company Irvine California USA; ^13^ Allergan Aesthetics, an AbbVie Company Madrid Spain

**Keywords:** BOTOX, combined treatment, infraorbital hollow, Juvéderm, periorbital area, rejuvenation, satisfaction

## Abstract

**Background:**

The periorbital region is among the first areas noticed in an individual, playing a crucial role in facial attractiveness and emotional expression.

**Aims:**

Open‐label study to evaluate the impact of mid and upper facial treatment with hyaluronic acid (HA) fillers and onabotulinumtoxinA (onabotA) on periorbital satisfaction and appearance.

**Patients/Methods:**

Eligible participants received ≥ 2 HA fillers (VYC‐15L, VYC‐17.5L, VYC‐20L) in the mid and upper face on Day 1, and VYC‐15L in the infraorbital hollow (IOH) on Day 30 if needed. Optional touch ups followed after 14 days. OnabotA was administered to glabellar lines (GL) and/or lateral canthal lines (LCL) on Day 60. The primary endpoint was the change from baseline in FACE‐Q Satisfaction with Eyes to Day 90. Additional effectiveness assessments and safety were monitored throughout.

**Results:**

Enrolled participants were 86.3% White and 13.7% Asian. FACE‐Q Rasch‐transformed scores increased from baseline to Day 90 by 55.5 points (*p* < 0.0001). Investigator‐assessed infraorbital appearance improved in 69.4% of participants at Day 30 (*p* = 0.063 vs. baseline) and in 100% at Day 90. Periorbital appearance improved in 91.7% (investigator) and 95.8% (participant) of participants at Day 30, reaching 100% for both at Day 90 (nominal *p* < 0.0001 vs. baseline). IOH severity gradually decreased, with 59.8% of participants showing minimal or no severity at Day 90 versus 0% at baseline, and infraorbital volume increased. No unexpected safety signals were observed.

**Conclusions:**

Combination of HA fillers and onabotA treatments improved eye satisfaction, periorbital and infraorbital appearance, IOH severity, and infraorbital volume, demonstrating direct and indirect treatment effects.

## 1 Introduction

Periorbital changes over time can lead to expressions that unintentionally convey anger, fatigue, or sadness, resulting in misinterpreted emotions and contributing to an aged appearance [1]. Several factors contribute to this aging effect, including changes in the orbital shape due to bone remodeling and resorption, which cause the eyes to appear smaller and more rounded. Fat loss and descent in areas such as the superomedial orbit and nasojugal groove leads to hollowing and the prominence of tear troughs. Additionally, the formation of lateral canthal lines (LCL) from repeated lateral orbicularis oculi muscle contractions, along with glabellar lines (GL) formed by the repeated corrugator supercilii and procerus muscles contractions, further adds to the aged appearance [2].

Rejuvenation of the periorbital area can enhance overall facial perception, contributing to improved psychosocial well‐being for the patient [3, 4]. However, direct treatment of this region is considered challenging due to its complex anatomy, concurrent deformities, and potential risk of complications [5]. Because facial anatomical regions are interconnected, alterations in neighboring areas can significantly affect periorbital esthetics [6, 7, 8]. The combination of malar fat ptosis with infraorbital volume loss has been associated with the development of infraorbital hollowing [7, 9]. Consequently, restoration of midface volume with soft‐tissue fillers has shown increased patient satisfaction with infraorbital appearance [6]. Additionally, weakening of the suspensory action of the frontalis muscle, coupled with the loss of support due to reduced fat volume in the temples and lateral forehead, can lead to eyebrow ptosis [10]. Filler application in the temple has shown lateral orbital and malar lifting effects [8, 11]. Treatment of forehead, eyebrows, lateral canthal region, eyelid cheek junction, and lateral cheek areas as one esthetic subunit has been suggested for optimal, natural results in eye rejuvenation [12].

OnabotulinumtoxinA (onabotA; BOTOX, VISTABEL, Allergan Aesthetics, an AbbVie company, Westport, Ireland) has demonstrated safety and efficacy in the treatment of upper facial lines [13, 14]. When combined with fillers, this approach can address muscle hyperactivity (onabotA) and volume deficits (fillers), both components of the aging process, yielding better outcomes than individual treatments [15, 16]. The combination of onabotA with hyaluronic acid (HA) fillers has also been associated with significant improvements in psychological well‐being and perceived age [4, 17]. This combined approach has become a standard of care and should be tailored to meet specific patient needs, considering factors like physiologic age, ethnic facial features, and cultural esthetic ideals [18].

Considering the impact of treatments on adjacent areas and the benefits of combining treatments, this study aimed to assess participants' satisfaction with the appearance of the periorbital area after treating the upper and mid‐face with various HA fillers, including VYC‐15L (Juvéderm VOLBELLA with Lidocaine; Allergan Aesthetics, an AbbVie company, Pringy, France), VYC‐17.5L (Juvéderm VOLIFT with Lidocaine Allergan Aesthetics, an AbbVie company, Pringy, France), and VYC‐20L (Juvéderm VOLUMA with Lidocaine; Allergan Aesthetics, an AbbVie company, Pringy, France), along with onabotA. The study also included a separate cohort analysis for Asian participants.

## 2 Materials and Methods

### 2.1 Study Design

This study was a 90‐day, prospective, open‐label, uncontrolled, and multicenter trial conducted at 8 sites in Belgium and Australia from January to October 2023. The study included an initial screening period from Day −14 to Day 1, followed by baseline assessments and initial treatment on Day 1. Subsequent treatments and posttreatment follow‐up visits occurred on Days 14, 30, 44, and 60. Participants returned for a final evaluation on Day 90 (study exit visit).

The study was designed, monitored, and conducted in accordance with the International Council for Harmonization (ICH) guidelines, and applicable regulations, and adhered to the principles outlined in the Declaration of Helsinki. Participants voluntarily signed and dated an informed consent prior to screening or the beginning of study procedures.

### 2.2 Treatment Administration

On Day 1, initial treatments were administered to the mid and upper face areas (e.g., eyebrows, temples, malar/zygomatic areas, periorbital fine lines), excluding the infraorbital hollow (IOH), using VYC‐15L, VYC‐17.5L, and/or VYC‐20L. Optional touch‐up treatments with the HA fillers for the treated areas were allowed on Day 14. If necessary, VYC‐15L was administered to the IOH on Day 30, with an optional touch‐up on Day 44. The maximum filler volumes allowed per participant for both initial and touch‐up treatments combined were: VYC‐15L up to 2 mL, VYC‐17.5L up to 4 mL, and VYC‐20L up to 4 mL. The choice of product, area, and injection volume for both initial and touch‐up treatments were determined by the investigator's clinical experience and participant esthetic goals, adhering to the product's label directions.

Participants received intramuscular injections of onabotA for GL (20 U, five injection points) and/or LCL (12 U per side in three injection points) at Day 60. There were no touch‐ups for onabotA.

### 2.3 Participants

Eligible participants were adults aged 40 to 65, who were dissatisfied with their eyes, measured by a baseline score of “Very dissatisfied” or “Somewhat dissatisfied” in at least 3 out of the 7 items of the FACE‐Q Satisfaction with Eyes scale. Eligible participants presented with minimal to extreme IOH on both sides, as indicated by a score between 1 (Minimal) to 4 (Extreme) on the Allergan Infraorbital Hollows Scale (AIHS) and needed treatment in at least 2 areas of the mid and/or upper face with at least 2 of the HA fillers, as assessed by the investigator. Additionally, eligible participants met at least 1 of the following criteria per area‐specific investigator‐assessed severity scales for static lines: Grade 2 or 3 (Moderate or Severe) on the Allergan Glabellar Lines Severity Scale (AGLSS) at maximum frown and/or Grade 2 or 3 (Moderate or Severe) on the Lateral Canthal Line Severity Scale (LCLSS) at maximum smile.

Key exclusion criteria included prior neurotoxin treatments in the mid and/or upper face for any indication within 12 months preceding the study entry or filler injections in these areas within the last 2 years.

### 2.4 Effectiveness Assessments

The primary effectiveness endpoint was the change from baseline to Day 90 in the FACE‐Q Satisfaction with Eyes score. This validated scale includes seven items, each scored on a 4‐point Likert scale ranging from “Very dissatisfied” to “Very satisfied.” [19] Individual item scores were summed and converted to a scale from 0 to 100, where higher scores indicate greater satisfaction.

Secondary endpoints included the investigator‐assessed responder rate on the global esthetic improvement scale (GAIS) for the infraorbital area and the responder rate on the GAIS for the periorbital area based on the individual assessments of the investigator and participant. The GAIS evaluates overall esthetic improvement with categories ranging from “Much worse” to “Much improved,” scored on a 5‐point scale from −2 to +2. A GAIS responder was defined as a participant who showed improvement in the overall esthetic assessment (responses of “Improved” or “Much improved”).

Additional endpoints encompassed the change from baseline in the severity of infraorbital hollowing, as assessed by the investigator using the AIHS, a validated 5‐point scale ranging from 0 (None) to 4 (Extreme) [20]. Changes in infraorbital volume were measured using three‐dimensional imaging technology. The investigator also evaluated the GL severity at maximum frown using the AGLSS, as well as the LCL severity at maximum smile using the LCLSS at all applicable visits. Both scales use a 4‐point rating system from 0 (None) to 3 (Severe).

### 2.5 Safety Assessments

In the safety assessments, participants reported injection site responses (ISRs) daily via electronic diaries for 14 days following filler treatments, detailing symptom incidence, maximum severity, and duration for both initial and touch‐up treatments. Adverse events were monitored throughout the study.

### 2.6 Statistical Analysis

A sample size of 64 participants was estimated to provide at least 97% power to detect a change from baseline to Day 90 in the overall FACE‐Q Satisfaction with Eyes score using a 1‐sample, 2‐sided *t* test at a 5% significance level. To account for an anticipated 20% dropout rate, the study aimed to enroll a minimum of 80 participants.

Effectiveness was analyzed in the evaluable study population, which consisted of all participants who met eligibility criteria at the screening visit, received study treatment, and had a baseline and Day 90 post‐treatment effectiveness assessment. The primary effectiveness endpoint was analyzed using a paired *t*‐test. Responder analyses for secondary endpoints were summarized using frequencies and percentages. Additionally, a *p* value and the corresponding 95% confidence interval (CI), based on the binomial exact test, were calculated to assess the statistical significance of the GAIS responder rate being greater than 60%. Additional endpoints were summarized using descriptive statistics for specified assessment visits. Responder status for additional endpoints was presented using frequencies, percentages, and 95% CI based on the exact binomial distribution.

The safety population consisted of all participants who met eligibility criteria at the screening visit and received any treatment product during the study. Safety assessments were reported using descriptive statistics.

## 3 Results

### 3.1 Participant Disposition and Demographics

Of the 82 participants screened, 73 were treated and completed the study. Of those, 72 participants were included in the evaluable population and 73 in the safety population. One treated participant was excluded from the evaluable set due to missing the Day 90 primary effectiveness assessment.

The mean age of the participants was 51.3 years (range, 41–66 years). The majority of participants were women (90.4%), and White (86.3%). The distribution of Fitzpatrick skin phototypes was as follows: I/II (46.6%), III/IV (52.1%), and V/VI (1.4%). Ten (13.7%) of the enrolled participants were Asian, with a majority being of Chinese ethnicity; all were women and had a mean age of 45.0 years (range, 41–61 years). Of the 10 Asian participants, 9 (90%) had a Fitzpatrick skin phototype IV, while the remaining participant had a phototype V.

At baseline, participants in the evaluable population exhibited Moderate (31.5%), Severe (49.3%), or Extreme infraorbital hollowing (19.2%). Among Asian participants, investigator assessments on the AIHS rated 50.0% as Moderate, 40.0% as Severe, and 10.0% as Extreme. Additionally, most participants in the evaluable population had Moderate (49.3%) or Severe (46.6%) GL, and Moderate (26.0%) or Severe (72.6%) LCL (Table 1). For Asian participants, 70.0% scored as Moderate and 30.0% as Severe on GL severity, while participants were evenly split with 50.0% each for Moderate and Severe on LCL severity (Table 1).

**TABLE 1:** Participant demographic and baseline characteristics.

Characteristics	Total (*N* = 73)	Asian cohort (*N* = 10)
Age, mean (SD), years	51.3 (6.86)	45.0 (6.25)
Sex, *n* (%)		
Female	66 (90.4)	10 (100.0)
Male	7 (9.6)	0 (0.0)
Race, *n* (%)		
Asian^a^	10 (13.7)	
White	63 (86.3)	
Fitzpatrick skin phototype, *n* (%)		
I/II	34 (46.6)	0 (0.0)
III/IV	38 (52.1)	9 (90.0)
V/VI	1 (1.4)	1 (10.0)
Baseline AIHS score, *n* (%)		
Minimal	0 (0.0)	0 (0.0)
Moderate	23 (31.5)	5 (50.0)
Severe	36 (49.3)	4 (40.0)
Extreme	14 (19.2)	1 (10.0)
Baseline AGLSS score, *n* (%)		
Minimal	3 (4.1)	0 (0.0)
Moderate	36 (49.3)	7 (70.0)
Severe	34 (46.6)	3 (30.0)
Baseline LCLSS score, *n* (%)		
Minimal	1 (1.4)	0 (0.0)
Moderate	19 (26.0)	5 (50.0)
Severe	53 (72.6)	5 (50.0)

Abbreviations: AGLSS, Allergan Glabellar Lines Severity Scale; AIHS, Allergan Infraorbital Hollows Scale; LCLSS, Lateral Canthal Line Severity Scale.

^a^ All Asian participants were of East Asian descent, primarily of Chinese ethnicity.

### 3.2 Treatment Administration

In the initial treatment, five participants (6.8%) received VYC‐15L, 70 (95.9%) received VYC‐17.5L, and 72 (98.6%) received VYC‐20L. For the optional touch‐up treatments, five participants (6.8%) received VYC‐15L, 28 (38.4%) received VYC‐17.5L, and 18 (24.7%) received VYC‐20L. In the IOH treatment, 66 participants (90.4%) received VYC‐15L and 25 (34.2%) had touch‐up treatments. The average total volume injected, including initial treatment and touch‐up, was 1.4 mL for VYC‐15L, 2.2 mL for VYC‐17.5L, and 2.8 mL for VYC‐20L (Table 2). All participants were treated with onabotA for GL (20 U) and LCL (12 U/side).

**TABLE 2:** Mean filler volume injected per facial area in initial and touch‐up treatments combined.

	VYC‐15L		VYC‐17.5L		VYC‐20L	
	*n*	Volume, mL	*n*	Volume, mL	*n*	Volume, mL
Total volume						
Asian cohort	10	1.2	9	2.0	10	2.9
Evaluable population	68	1.4	72	2.2	72	2.8
Temple						
Asian cohort	0	—	7	1.6	6	1.4
Evaluable population	2	0.8	32	1.2	50	1.2
Cheek						
Zygomaticomalar region						
Asian cohort	0	—	0	—	3	1.2
Evaluable population	0	—	10	0.9	64	1.2
Anteromedial region						
Asian cohort	0	—	6	0.9	8	1.6
Evaluable population	1	0.5	40	1.0	53	1.0
Submalar region						
Asian cohort	0	—	1	1.0	1	1.0
Evaluable population	0	—	22	1.1	14	0.8
Eyebrow						
Asian cohort	0	—	0	—	3	0.8
Evaluable population	1	0.6	13	0.7	7	0.7
Forehead						
Asian cohort	—	—	—	—	—	—
Evaluable population	0	—	19	1.3	0	—
Periorbital						
Asian cohort	—	—	—	—	—	—
Evaluable population	6	0.7	7	1.2	0	—
Infraorbital						
Asian cohort	10	1.2	0	—	0	—
Evaluable population	66	1.4	0	—	0	—

Among the 10 Asian participants, nine received VYC‐17.5L and all 10 received VYC‐20L during the initial treatment. One participant received a touch‐up with VYC‐20L. All 10 participants underwent IOH treatment with VYC‐15L, with only one requiring a touch‐up. The total volumes injected for the Asian participants were comparable to those for the entire population (Table 2). Similarly, all Asian participants received onabotA treatment for both GL and LCL.

Regarding the facial areas treated with each filler before touch‐up treatments, 97.1% of VYC‐15L injections targeted the infraorbital area, mostly at a supraperiosteal depth. VYC‐17.5L was more often administered to the temples (35.7%) and the cheek anteromedial region (54.3%) using subcutaneous or supraperiosteal injections. Additionally, VYC‐20L was predominantly administered to the temples (63.9%) as well as the zygomaticomalar and anteromedial regions of the cheek (87.5% and 73.6%, respectively), most commonly in the supraperiosteal plane. For Asian participants, filler injections mirrored those in the entire population.

Touch‐up treatments focused on the most commonly treated areas for each filler, with VYC17.5L injections concentrating on the submalar cheek in addition to the temple for the evaluable population. Touch‐up treatments were less frequent in the Asian cohort.

The majority of injections for VYC‐15L and VYC‐17.5L were administered with a 25 G cannula, and injections for VYC‐20L were more frequently administered with a 27 G needle. Touch up treatments were administered similarly.

### 3.3 Effectiveness Endpoints

#### 3.3.1 FACE‐Q Satisfaction With Eyes

**FIGURE 1:**
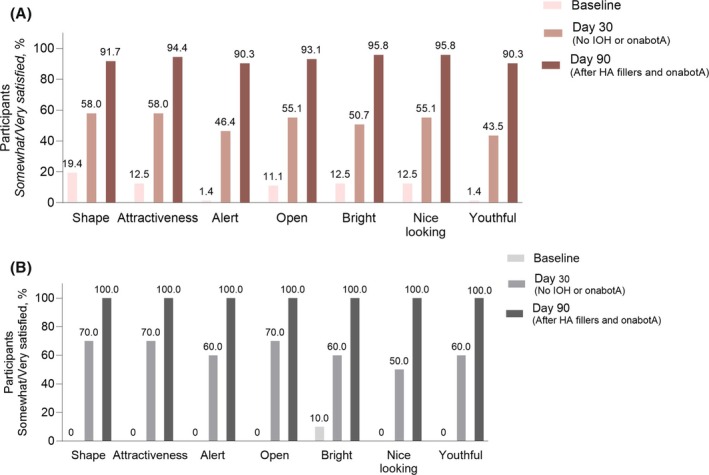
Individual concepts of FACE‐Q Satisfaction with Eyes scale in (A) the evaluable population and (B) the Asian cohort. HA, hyaluronic acid; IOH, infraorbital hollow; onabotA, onabotulinumtoxinA.

For the primary effectiveness endpoint, the overall FACE‐Q Rasch‐transformed score increased from a mean of 23.0 (95% CI, 19.27–26.81) at baseline to 78.6 (95% CI, 74.18–82.96) at Day 90, resulting in a significant mean increase of 55.5 points (95% CI, 49.31–61.74; *p* < 0.0001). In the Asian cohort, the overall FACE‐Q Rasch‐transformed score change from baseline to Day 90 was 67.7 points (95% CI, 59.93–75.47). Participant satisfaction gradually increased over time in each item of the FACE‐Q Satisfaction with Eyes assessment. At baseline, over 80% of participants were somewhat/very dissatisfied across all seven evaluated items. At Day 30, before treating the IOH or using onabotA, 58.0% of participants were somewhat/very satisfied with the attractiveness of their eyes, 55.1% with how open their eyes look, and 46.4% with how alert their eyes look, compared to 12.5%, 11.1%, and 1.4% at baseline. Satisfaction continued increasing until Day 90, with over 90% of participants expressing satisfaction across all evaluated concepts (Figure 1A). Satisfaction with the eyes also improved over time among Asian participants. At baseline, 100.0% of Asian participants were somewhat/very dissatisfied with 6 of the 7 items, and 90.0% were somewhat/very dissatisfied with eye brightness; by Day 90, 100.0% were somewhat/very satisfied with all items (Figure 1B).

#### 3.3.2 GAIS Assessments

**FIGURE 2:**
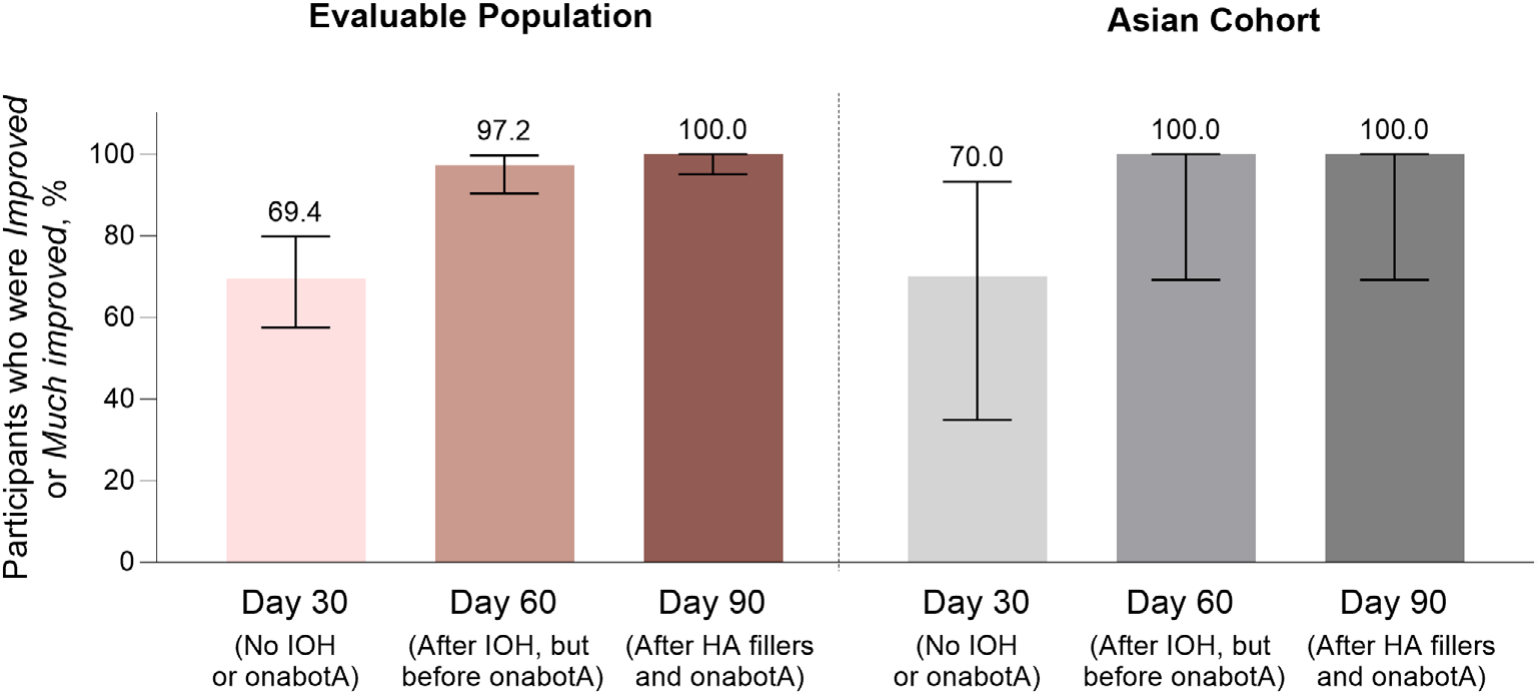
Global esthetic improvement of the infraorbital area, as assessed by the investigator. Error bars indicate the 95% CIs. HA, hyaluronic acid; IOH, infraorbital hollow; onabotA, onabotulinumtoxinA.

**FIGURE 3:**
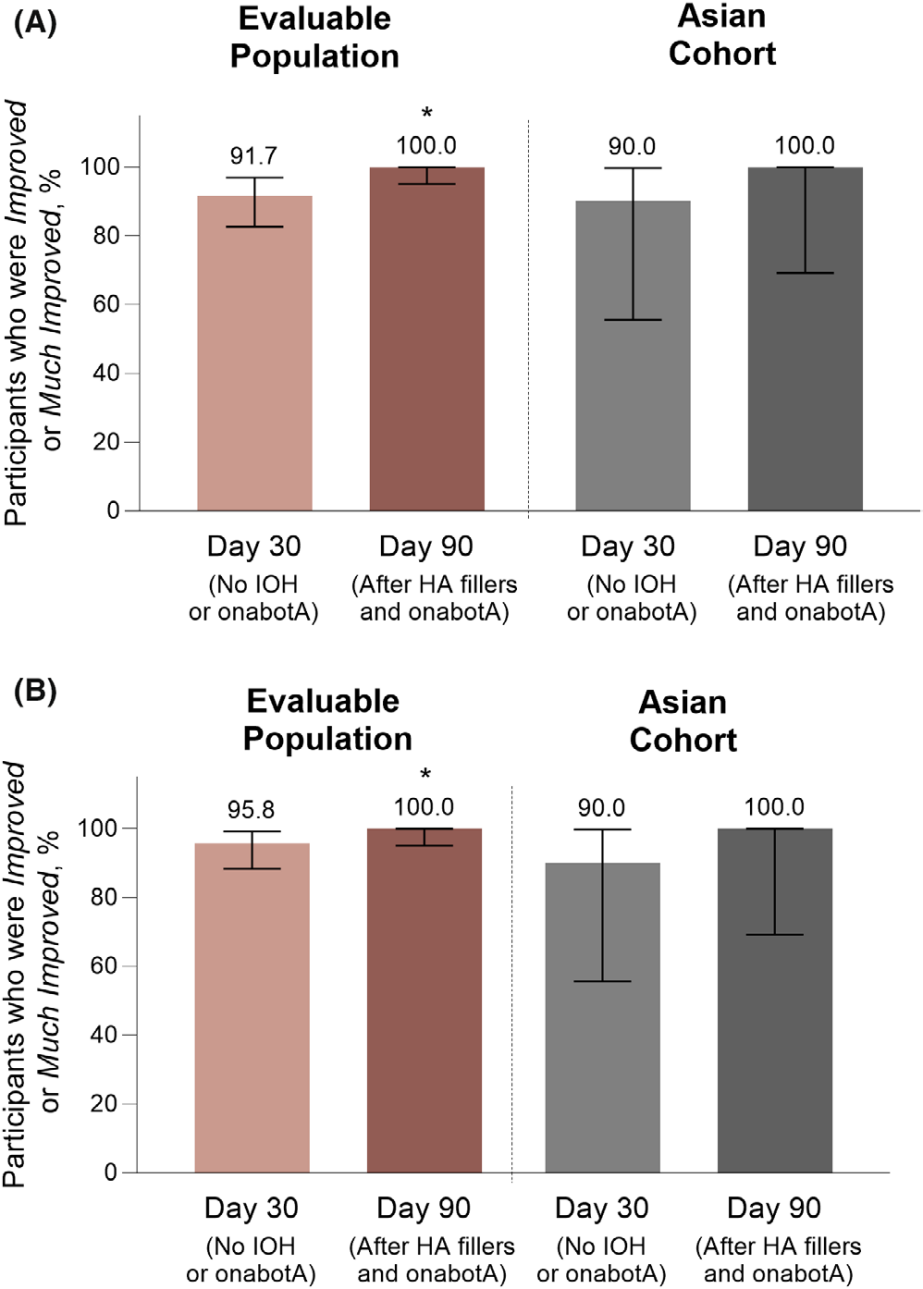
Global esthetic improvement of the periorbital area, as assessed by (A) the investigator and (B) the participants. Error bars indicate the 95% CIs. *Nominal *P* < 0.0001 versus baseline. HA, hyaluronic acid; IOH, infraorbital hollow; onabotA, onabotulinumtoxinA.

GAIS assessments showed gradual improvements in both the infraorbital and periorbital areas after subsequent treatments among both the evaluable and the Asian populations (Figures 2 and 3A,B). For the infraorbital area, the investigator‐assessed GAIS showed a high degree of improvement by Day 30, when only the mid and upper face (excluding the IOH) had been treated with fillers. Improvement at Day 30 was seen in 69.4% of participants (95% CI, 57.5%–79.8%; *p* = 0.063 vs. baseline) in the evaluable population. The percentage of participants with improvement in the infraorbital area rose to 97.2% (95% CI, 90.3%–99.7%) at Day 60 and reached 100.0% (95% CI, 95.0%–100.0%) by Day 90 (Figure 2).

In the periorbital area at Day 30, investigator GAIS assessments showed improvement in 91.7% (95% CI, 82.7%–96.9%) of participants in the evaluable population. By Day 90, 100.0% (95% CI, 95.0%–100.0%; nominal *p* < 0.0001 vs. baseline) of participants were rated as “Improved” or “Much improved” (Figure 3A). Similar results were observed when participants assessed global esthetic improvement, with 95.8% (95% CI, 88.3%–99.1%) of participants in the evaluable population achieving “Improved” or “Much improved” periorbital appearance at Day 30. The percentage of participants with improved periorbital appearance reached 100.0% at Day 90 (95% CI, 95.0%–100.0%; nominal *p* < 0.0001 vs. baseline) (Figure 3B).

#### 3.3.3 AIHS

Severity of the IOH gradually decreased throughout the study. The overall mean AIHS score decreased from 2.9 (95% CI, 2.71–3.04) at baseline to 2.3 (95% CI, 2.15–2.55) at Day 30, with a mean change of −0.5 (95% CI, −0.73, −0.32), following filler injections in the mid and upper face (excluding the IOH). The mean score was further reduced to 1.3 (95% CI, 1.14–1.50) at Day 60, with a mean change from baseline of −1.6 (95% CI, −1.75, −1.36). By Day 90, the overall mean score remained at 1.3 (95% CI, 1.09–1.49), showing sustained improvement in IOH severity.

**FIGURE 4:**
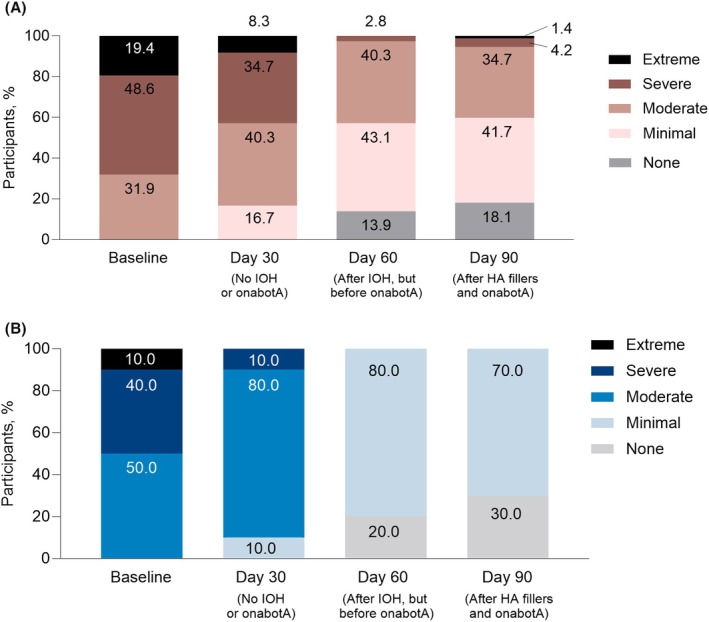
Severity category distribution on the Allergan Infraorbital Hollow Severity scale for (A) the evaluable population and (B) the Asian cohort. HA, hyaluronic acid; IOH, infraorbital hollow; onabotA, onabotulinumtoxinA.

When examining the percentage of participants in each AIHS category, 31.9% of participants were Moderate, 48.6% were Severe, and 19.4% were Extreme at baseline. Scores improved at Day 30 after filler treatment of the mid and upper face (excluding the IOH), with a rise in participants scoring Minimal or Moderate (16.7% and 40.3%, respectively) and a decrease in those scoring Severe or Extreme (34.7% and 8.3%, respectively). At Day 90, the majority of participants were Minimal (41.7%) or Moderate (34.7%), with only 1.4% and 4.2% in the Extreme and Severe categories, respectively; additionally, 18.1% of participants presented no symptoms. Figure 4A illustrates the progression of IOH severity to milder or nonexistent over time in the evaluable population, while Figure 4B depicts the decline in Asian participants.

#### 3.3.4 Infraorbital Volume

The 3D volumetric analysis showed a mean increase in infraorbital volume for both the left and right eyes of 0.5 cc (95% CI, 0.34–0.61 cc) and 0.4 cc (95% CI, 0.23–0.51 cc) respectively, from baseline to Day 30, following filler treatment of the mid and upper face, excluding the IOH. By Day 60, after direct treatment of the IOH, further volume gains were observed on both eyes, with mean increases from baseline of 1.1 cc (left; 95% CI, 0.95–1.26 cc) and 1.0 cc (right; 95% CI, 0.85–1.19 cc). Volumetric changes were sustained from Day 60 to Day 90 after onabotA treatment. The infraorbital volume in Asian participants showed similar results.

#### 3.3.5 AGLSS and LCLSS

A decrease in GL severity was reported by investigators after onabotA treatment using the AGLSS. There was a mean reduction of −2.0 (95% CI, −2.17, −1.83) points from a baseline mean score of 2.4 (95% CI, 2.29–2.57) to a score of 0.4 (95% CI, 0.29–0.57) at Day 90 in the evaluable population. The AGLSS score in Asian participants also decreased by a mean of −2.0 (95% CI, −2.34, −1.66) points from baseline to Day 90.

**FIGURE 5:**
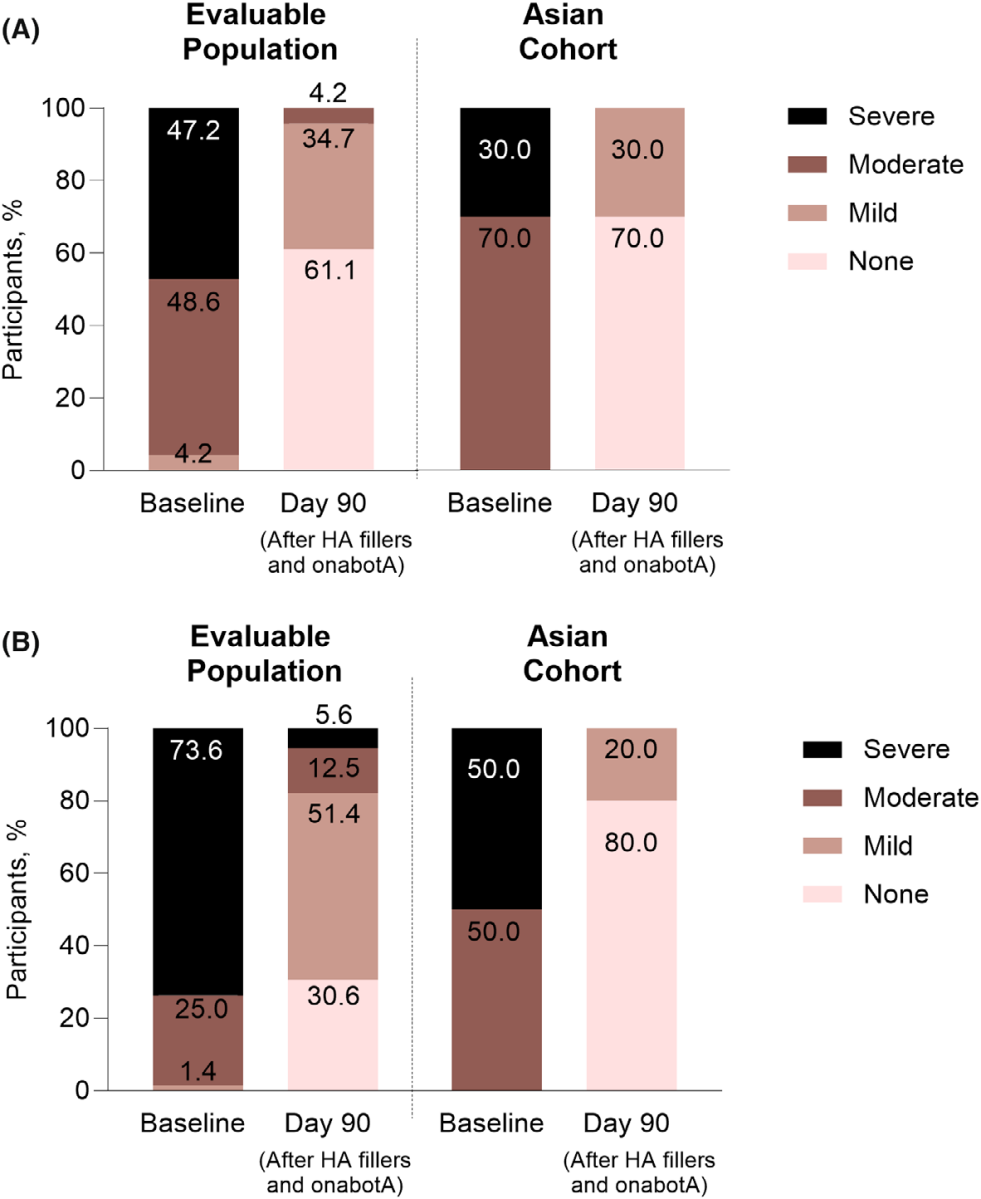
Severity category distribution on the (A) Allergan Glabellar Line Severity Scale and (B) the Lateral Canthal Line Severity Scale. HA, hyaluronic acid; IOH, infraorbital hollow; onabotA, onabotulinumtoxinA.

When examining percentages of participants per response category, 48.6% of participants in the evaluable population were scored Moderate and 47.2% Severe at baseline; at Day 90, these percentages decreased to 4.2% and 0.0%, respectively (Figure 5A). Among Asian participants, the majority (70.0%) were scored Moderate at baseline and improved to none at Day 90 (Figure 5A).

On the LCLSS, a mean decrease of −1.8 (95% CI, −1.98, −1.61) points from a baseline score of 2.7 (95% CI, 2.61–2.84) to a score of 0.9 points (95% CI, 0.74, 1.12) at Day 90 was noted by investigators in the evaluable population. The Asian cohort had a mean decrease of −2.3 (95% CI, −2.65, −1.95) points from baseline to Day 90. In the evaluable population, the percentages of participants scored as Moderate and Severe dropped from 25.0% and 73.6% at baseline to 12.5% and 5.6% after treatment (Figure 5B). Asian participants were scored either Moderate or Severe (50.0% each) at baseline, but the majority (80.0%) were scored none at Day 90 (Figure 5B).

**FIGURE 6:**
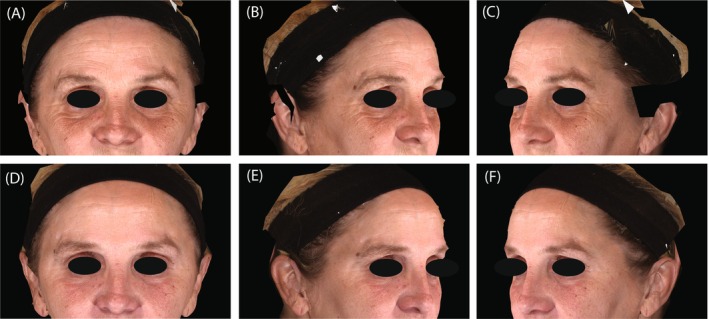
Representative photographs of the periorbital area before (A–C) and 90 days after (D–F) sequential treatment with hyaluronic acid fillers and onabotulinumtoxinA. A 55‐year‐old female participant received treatment in the temples (2.0 mL of VYC‐17.5L and 0.5 mL of VYC‐20L), cheeks (2.0 mL of VYC‐17.5L, and 3.5 mL of VYC‐20L), and the infraorbital hollow (1.45 mL of VYC‐15L). She also received 20 units of onabotulinumtoxinA in the glabellar lines and 12 units per side in the lateral canthal lines. At baseline, her assessments included a FACE‐Q Satisfaction with Eyes Rasch‐transformed score of 0, Allergan Infraorbital Hollow Scale (AIHS) score of 3 (Severe) on both sides, Allergan Glabellar Line Severity Scale (AGLSS) score of 2 (Moderate), and Lateral Canthal Line Severity Score (LCLSS) score of 3 (Severe). At Day 90, the FACE‐Q score improved by 59 points to 59, and the AIHS achieved a score of 1 (Minimal) on the left side and 0 (None) on the right side; the AGLSS decreased to 0 (None) and the LCLSS to 2 (Moderate).

**FIGURE 7:**
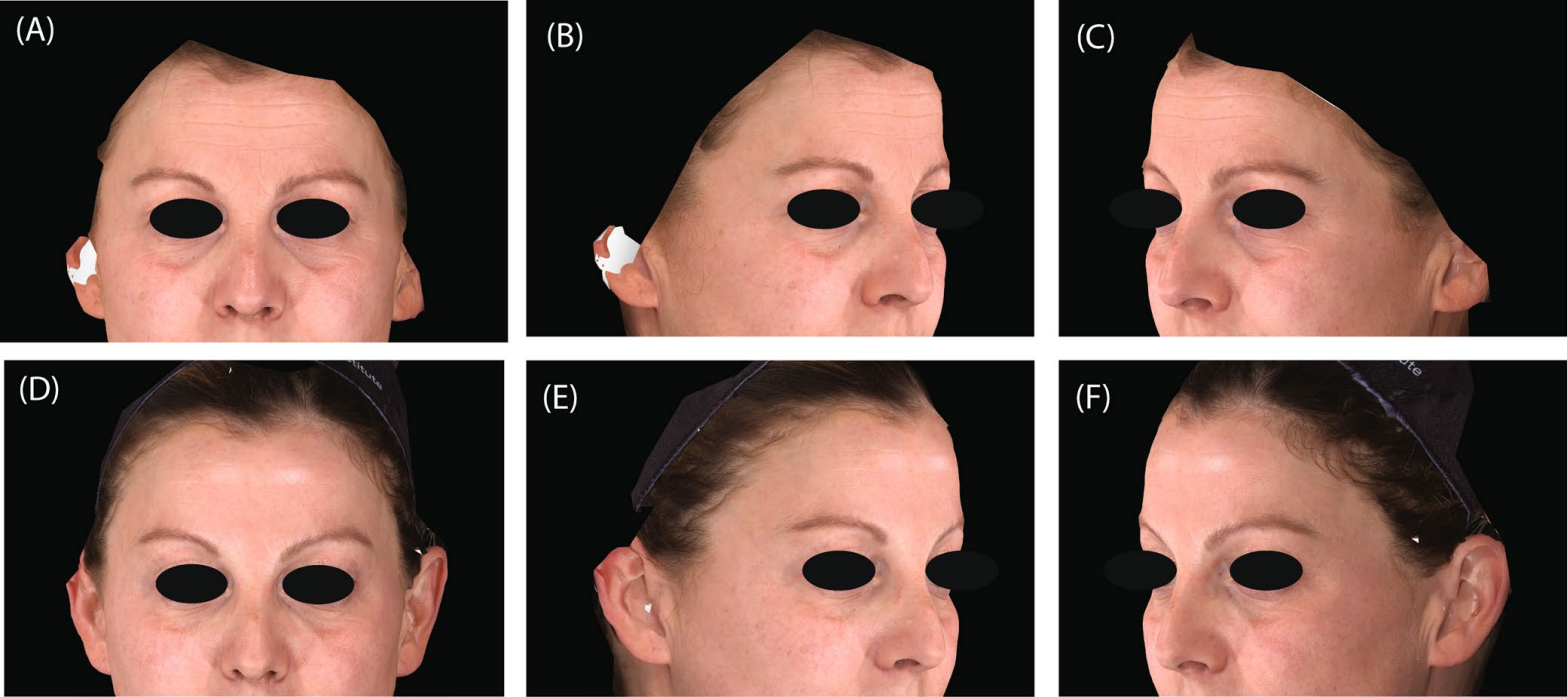
Representative photographs of the periorbital area (A–C) and 90 days after (D–F) sequential treatment with hyaluronic acid fillers and onabotulinumtoxinA. A 42‐year‐old female participant received treatment in the cheeks (2.0 mL of VYC‐17.5L and 1.0 mL of VYC‐20L), temples (1.0 mL of VYC‐20L), and infraorbital hollow (0.6 mL of VYC‐15L). She also received 20 units of onabotulinumtoxinA in the glabellar lines and 12 units per side in the lateral canthal lines. At baseline, her assessments included a FACE‐Q Satisfaction with Eyes Rasch‐transformed overall score of 35, Allergan Infraorbital Hollow Scale (AIHS) score of 3 (Severe) on both sides, and both Allergan Glabellar Line Severity Scale (AGLSS) and Lateral Canthal Line Severity Score (LCLSS) scores of 3 (Severe). At Day 90, the FACE‐Q score improved by 37 points to 72, and the AIHS achieved a score of 1 (Minimal) on both sides; the AGLSS decreased to 0 (None) and the LCLSS to 1 (Mild).

Figures 6 and 7 present representative photographs illustrating the improvement in the periorbital area before and after treatments (Day 90).

### 3.4 Safety

For the initial and touch‐up treatments, most ISRs were mild in severity and resolved within 1–3 days. The most common ISRs were tenderness to touch and swelling. The incidence of ISRs was higher for initial treatment and lower for all subsequent visits. Similar trends were reported for Asian participants.

Regarding treatment‐emergent adverse events (TEAEs), 15 treated participants reported a total of 32 events after filler treatments. The most frequently reported TEAEs (≥ 3% incidence) were injection site edema (10 events in five participants [6.8%]), and injection site pain (7 events in four participants [5.5%]). Most TEAEs were mild in severity.

In terms of treatment‐related TEAEs, nine participants (12.3%) experienced a total of 19 events after filler treatments, the most frequent being edema (6.8%) and pain (5.5%), all at the injection site. One event of moderate headache was reported with VYC‐20L injection into the temple (Table 3). Most treatment‐related TEAEs with the HA fillers were mild in severity and began within 1 week of treatment for VYC17.5L and VYC‐20L, and within 15–30 days for VYC‐15L. Of the 19 events, 11 resolved within a week. At the end of the study, eight events of mild edema persisted in four participants, with events affecting the left and right sides of the midface in the same participant counted separately; none required medical intervention during the study period. OnabotA led to six treatment‐related TEAEs of edema at the injection site in three participants (4.1%) (Table 3). These events were all mild in severity, and most began within 15 to 30 days of onabotA treatment. No treatment‐related TEAEs associated with filler treatments or onabotA were reported for the Asian cohort.

**TABLE 3:** Summary of treatment‐related adverse events in the safety population.

Treatment‐related TEAE, *n* (%)^a^	All fillers (*N* = 73)		OnabotA (*N* = 73)	
	Participants *n* (%)	Events	Participants *n* (%)	Events
Any treatment‐related TEAE	9 (12.3)	19	3 (4.1)	6
Injection‐site edema	5 (6.8)	10	3 (4.1)	6
Injection‐site pain	4 (5.5)	7	0 (0.0)	0
Injection‐site bruising	1 (1.4)	1	0 (0.0)	0
Headache	1 (1.4)	1	0 (0.0)	0

Abbreviations: OnabotA, onabotulinumtoxinA; TEAE, treatment‐emergent adverse event.

^a^ No treatment‐related TEAEs were reported for the Asian cohort.

One participant reported two serious TEAEs of sinusitis, both mild in severity and not related to the study treatment. There were no discontinuations due to TEAEs, and no deaths were reported.

## 4 Discussion

Following the pandemic, increased attention on exposed facial areas due to mask coverage of the lower face has enhanced patient awareness of the role of the periorbital region in perceived attractiveness [21]. The increased prevalence of videoconferencing has also led to heightened dissatisfaction with facial appearance, elevating concerns about specific features, such as the eyes [22]. This post‐marketing, open‐label, multicenter study showed a high level of improvement on the esthetics of the periorbital area after sequential treatment of the mid and upper face with a combination of HA fillers (VYC‐15L, VYC‐17.5L, and/or VYC‐20L) and onabotA using investigator‐assessed scales and multiple validated PRO measures. The primary endpoint of the study was met, with FACE‐Q scores rising significantly from baseline to Day 90 (*p* < 0.0001), Additionally, a global esthetic improvement (responses of “Improved” or “Much improved”) was achieved at Day 90 in the infraorbital area, and in the periorbital area (nominal *p* < 0.0001 vs. baseline). Investigators followed the approved product indications and volumes but used their expertise to tailor treatments to each participant's unique characteristics and needs, reflecting the approach used in real‐world clinical practice.

In this study, improvements in the periorbital area were observed as additional products and treatment areas were included. At Day 30, FACE‐Q Satisfaction with Eyes scores doubled following facial treatment of temples, forehead, eyebrows, periorbital lines, and/or cheeks with VYC‐15L, VYC‐17.5L, and/or VYC‐20L. By Day 90, after IOH treatment (VYC‐15L) and onabotA administration, FACE‐Q Satisfaction with Eyes scores increased threefold from baseline (*p* < 0.0001). Incremental gains were also observed across all individual concepts of the FACE‐Q Satisfaction with Eyes scale. Baseline satisfaction rates (participants somewhat/very satisfied) for all concepts was < 20%, with alert and youthful eyes scoring the lowest at 1.4%. At Day 30, after treating the mid and upper face with HA fillers, satisfaction levels increased over 3 times (range, 3.0–33.1 times) across all concepts compared to baseline. At Day 90, following direct IOH treatment and/or onabotA, satisfaction nearly doubled again (range, 1.6–2.1 times) compared to Day 30. Notably, satisfaction with alert and youthful eyes increased 33.1 times at Day 30 and 64.5 times at Day 90 compared to baseline. When evaluating global esthetic improvement in the periorbital area, GAIS scores at Day 30 exceeded 90% in both investigator and participant assessments. By Day 90, following all treatments, the periorbital area was improved in 100% of participants according to investigators and participants (nominal *p* < 0.0001 vs. baseline). Overall, these findings are consistent with existing literature, highlighting increased participant satisfaction and esthetic improvements following comprehensive facial treatments [15, 17]. However, to our knowledge, this study is the first to report on outcomes observed as products and treatment areas were sequentially included.

Furthermore, IOH improvements were observed following treatment of the mid and upper face with HA fillers, even though the IOH was not directly targeted. Investigator‐assessed GAIS responder rates for the infraorbital area surpassed 65% by Day 30. There was a reduction in AIHS scores (−0.5 points from baseline to Day 30), and 3D volumetric analysis showed an increase in infraorbital volume (0.5 cc on the left and 0.4 cc on the right). Previous studies indicated that treating the midface can contribute to enhance the esthetic appearance of the infraorbital area [6, 7]. The IOH improvements observed with mid and upper face treatment were followed by further changes after direct IOH treatment with VYC‐15L and onabotA, as evidenced by the shift of severity on the AIHS and 3D volumetric analysis, which showed sustained hollowing improvement and infraorbital volume increases from Day 30 to Day 90.

The investigator‐assessed GAIS score for the infraorbital area reported in this study following direct IOH treatment was comparable to the GAIS scores reported in previous HA filler studies targeting the IOH directly [23, 24]. However, the total median volume of VYC‐15L used for direct IOH treatment in this study was lower. In the current study, the total median VYC‐15L volume injected into the IOH, including initial treatment and touch‐ups, was 1.1 mL, while in a previous study that treated IOH alone, the total median VYC‐15L volume administered was 2.1 mL [24]. Reducing the filler volume may decrease the incidence of adverse events typically associated with IOH filler injections, such as edema [25]. Applying small filler aliquots in a staged approach is considered best practice for IOH rejuvenation [25]. These results emphasize the importance of considering both the target facial area and the adjacent anatomical regions to reduce injected volume and hence the risk of overcorrection and complications. Additionally, these findings may guide clinical practice in esthetics by supporting a sequential approach to filler administration, initiating treatment in adjacent facial areas before addressing the IOH.

Periorbital esthetics are a key consideration for Asian patients seeking to optimize their unique facial characteristics [26]. Anatomically, Asian patients exhibit a flatter malar area than Caucasian patients due to medial maxilla retrusion and a predisposition to “hooded” eyelids resulting from orbital rim retrusion and shallow orbits [27, 28]. These characteristics can accentuate IOHs and mid‐cheek volume deficits, which may emerge by the second decade in Asian patients, leading to a tired or prematurely aged appearance [28]. In this study, combined filler and onabotA treatment in the mid and upper face was followed by improvements in periorbital area esthetics among Asian participants, similar to the evaluable population. Indirect IOH improvements were observed after mid and upper face filler treatment, with additional gains seen after direct IOH intervention. All Asian participants received IOH treatment with VYC‐15L, and the median injected filler volumes were similar to the entire population. VYC‐17.5L and VYC‐20L were mainly used in the temples and anteromedial cheek region, reflecting ethnic structural features perceived as deficiencies. These results underscore the importance of a tailored approach to facial esthetics that respects anatomical nuances and cultural differences in facial esthetics.

Sequential administration of HA fillers and onabotA had a safety profile consistent with that of each individual product [29, 30, 31, 32]. ISRs were closely monitored, with most being mild in severity and transient for both initial and touch up treatments. In the Asian cohort, ISRs mirrored the overall population and decreased in subsequent visits. No treatments were discontinued due to adverse events, and there were no reports of serious treatment‐related adverse events. In Asian participants, no treatment‐related TEAEs were reported.

Edema was one of the most commonly reported treatment‐related TEAEs, with 8 cases ongoing at the end of the study; all were mild in severity and required no intervention. Edema is a frequently observed complication following treatment of the infraorbital region and tear trough [33, 34]. Comprehensive assessment of patient characteristics and anatomical considerations, along with appropriate filler selection and precise injection technique, may mitigate the risk of edema [25, 35]. Individuals with a history of fluid retention or preexisting malar or eyelid edema may be more susceptible to post‐treatment edema [25, 33, 35]. In the treatment of the infraorbital region, fillers with low to medium elasticity (G'), low cohesivity, and low water uptake are preferred to minimize the risk of swelling and ensure ease of spreadability [36]. A staged approach utilizing smaller filler volumes across multiple sessions and aiming for undercorrection is recommended [25]. Persistent edema can be resolved by administering hyaluronidase to dissolve the HA filler [25, 33, 35].

This study has some limitations. It was conducted in only two countries, Belgium and Australia, and primarily involved a female sample, which may restrict the generalizability of the findings. Given the multiproduct approach of the study design, it is not possible to determine the specific contribution of individual treatment components. In addition, the lack of a control group limits the ability to attribute observed outcomes solely to the administered interventions. Some assessments were performed by the injectors, possibly introducing bias in investigator‐assessed outcomes, despite this being the norm in clinical practice. The open‐label design may have also contributed to bias in the outcomes. Furthermore, the subgroup analysis was exploratory, and while differences from baseline to postinjection may appear meaningful, these differences could not be statistically tested.

In conclusion, the combined use of HA fillers and onabotA was associated with improvements in periorbital esthetics, while maintaining a safety profile comparable to each individual product. In this real‐world study, mid and upper facial (excluding IOH) filler treatments contributed to indirect enhancements in the periorbital region by Day 30, emphasizing the value of addressing interconnected facial areas to potentially reduce the need for direct intervention. Treatment benefits continued to increase through Day 90 after direct IOH infraorbital interventions and onabotA administration. This study, through its integrated approach, supports the potential for combined modalities to boost patient satisfaction and meet individual esthetic goals when treating the periorbital area.

## Author Contributions

Study design: Carola de la Guardia; Study investigators: Samira Baharlou, Sarah Boxley, Pierre Cuvelier, Gregory J. Goodman, Frank Lin, Cara McDonald, Joan Vandeputte, Ivar Van Heijningen; Enrolled patients: Samira Baharlou, Sarah Boxley, Pierre Cuvelier, Gregory J. Goodman, Frank Lin, Cara McDonald, Joan Vandeputte, Ivar Van Heijningen; Collection and assembly of data: All authors; Data analysis: Carola de la Guardia, Smita Chawla, Traci Baker; Data interpretation: All authors; Manuscript review and revisions: All authors; Final approval of manuscript: All authors.

## Ethics Statement

The study was conducted in accordance with the protocol, Operations Manual, International Council for Harmonization (ICH) guidelines, applicable regulations, and guidelines governing clinical study conduct and the ethical principles that have their origin in the Declaration of Helsinki, as well as ISO standards for investigational devices. The protocol, informed consent form(s), recruitment materials, and all participant materials were reviewed and approved by an Independent Ethical Committee. All participants provided written informed consent before study enrollment.

## Consent

Participants consented to publication of photographs with their eyes masked.

## Conflicts of Interest

Allergan Aesthetics, an AbbVie company, funded this study and participated in the design, research, analysis, data collection, interpretation of data, and the review and approval of the publication. All authors had access to relevant data and participated in the drafting, review, and approval of this publication. No honoraria or payments were made for authorship. Financial arrangements of the authors with companies whose products may be related to the present report are listed as declared by the authors: Gregory J. Goodman is an investigator and advisory board member for Allergan Aesthetics, an AbbVie company. Pierre Cuvelier is an investigator for Allergan Aesthetics, an AbbVie company, and faculty member of the Allergan Medical Institute. Frank Lin is an investigator for Allergan Aesthetics, an AbbVie company. Cara McDonald is an investigator for Allergan Aesthetics, an AbbVie company. Sarah Boxley is an investigator, consultant, and advisory board member for Allergan Aesthetics, an AbbVie company. Joan Vandeputte is an investigator for Allergan Aesthetics, an AbbVie company, consultant for Merz and Advanced Aesthetic Technologies, and holds limited stock options for Advanced Aesthetic Technologies. Ivar Van Heijningen is an investigator and speaker for Allergan Aesthetics, an AbbVie company, and member of the Allergan Medical Institute. Samira Baharlou is an investigator, speaker, and advisory board member for Allergan Aesthetics, an AbbVie company, speaker and trainer for the Allergan Medical Institute, and speaker for Galderma; she has received travel grants from Allergan Aesthetics, and AbbVie company, and Galderma, and research grants from Galderma. Traci Baker, Smita Chawla, and Carola de la Guardia are employees of AbbVie and may own AbbVie stock.

## Data Availability

AbbVie is committed to responsible data sharing regarding the clinical trials we sponsor. This includes access to anonymized, individual, and trial‐level data (analysis data sets), as well as other information (e.g., protocols, clinical study reports, or analysis plans), as long as the trials are not part of an ongoing or planned regulatory submission. This includes requests for clinical trial data for unlicensed products and indications. These clinical trial data can be requested by any qualified researchers who engage in rigorous, independent, scientific research and will be provided following review and approval of a research proposal, Statistical Analysis Plan (SAP), and execution of a Data Sharing Agreement (DSA). Data requests can be submitted at any time after approval in the US and Europe and after acceptance of this manuscript for publication. The data will be accessible for 12 months, with possible extensions considered. For more information on the process or to submit a request, visit the following link: https://vivli.org/ourmember/abbvie/ then select “Home.”

## References

[1] R. Fitzgerald , “Contemporary Concepts in Brow and Eyelid Aging,” Clinics in Plastic Surgery 40, no. 1 (2013): 21–42, 10.1016/j.cps.2012.08.005. 23186754

[2] A. Swift , S. Liew , S. Weinkle , J. K. Garcia , and M. B. Silberberg , “The Facial Aging Process From the “Inside out”,” Aesthetic Surgery Journal 41, no. 10 (2021): 1107–1119, 10.1093/asj/sjaa339. 33325497 PMC8438644

[3] K. R. Beer , H. Julius , M. Dunn , and F. Wilson , “Remodeling of Periorbital, Temporal, Glabellar, and Crow's Feet Areas With Hyaluronic Acid and Botulinum Toxin,” Journal of Cosmetic Dermatology 13, no. 2 (2014): 143–150, 10.1111/jocd.12082. 24910278

[4] J. L. Cohen , A. Rivkin , S. Dayan , et al., “Multimodal Facial Aesthetic Treatment on the Appearance of Aging, Social Confidence, and Psychological Well‐Being: HARMONY Study,” Aesthetic Surgery Journal 42, no. 2 (2022): NP115–NP124, 10.1093/asj/sjab114. 33751048 PMC8756087

[5] P. H. Peng and J. H. Peng , “Treating the Tear Trough: A New Classification System, a 6‐Step Evaluation Procedure, Hyaluronic Acid Injection Algorithm, and Treatment Sequences,” Journal of Cosmetic Dermatology 17, no. 3 (2018): 333–339, 10.1111/jocd.12514. 29504668

[6] J. Few , S. E. Cox , D. Paradkar‐Mitragotri , and D. K. Murphy , “A Multicenter, Single‐Blind Randomized, Controlled Study of a Volumizing Hyaluronic Acid Filler for Midface Volume Deficit: Patient‐Reported Outcomes at 2 Years,” Aesthetic Surgery Journal 35, no. 5 (2015): 589–599, 10.1093/asj/sjv050. 25964628 PMC4482214

[7] D. A. Glaser , V. Lambros , J. Kolodziejczyk , A. Magyar , K. Dorries , and C. J. Gallagher , “Relationship Between Midface Volume Deficits and the Appearance of Tear Troughs and Nasolabial Folds,” Dermatologic Surgery 44, no. 12 (2018): 1547–1554, 10.1097/DSS.0000000000001684. 30379685

[8] R. Haidar , M. D. D. Freytag , K. Frank , et al., “Quantitative Analysis of the Lifting Effect of Facial Soft‐Tissue Filler Injections,” Plastic and Reconstructive Surgery 147, no. 5 (2021): 765e–776e, 10.1097/PRS.0000000000007857. 33890889

[9] S. R. Coleman and R. Grover , “The Anatomy of the Aging Face: Volume Loss and Changes in 3‐Dimensional Topography,” Aesthetic Surgery Journal 26, no. 1S (2006): S4–S9, 10.1016/j.asj.2005.09.012. 19338976

[10] V. Bertucci , J. D. Carruthers , D. D. Sherman , C. J. Gallagher , and J. Brown , “Integrative Assessment for Optimizing Aesthetic Outcomes When Treating Glabellar Lines With Botulinum Toxin Type A: An Appreciation of the Role of the Frontalis,” Aesthetic Surgery Journal 43, no. Suppl 1 (2023): S19–S31, 10.1093/asj/sjac267. 36322138 PMC10638666

[11] G. Casabona , K. Frank , N. Moellhoff , et al., “Full‐Face Effects of Temporal Volumizing and Temporal Lifting Techniques,” Journal of Cosmetic Dermatology 19, no. 11 (2020): 2830–2837, 10.1111/jocd.13728. 32946624

[12] M. Pascali and O. Massarelli , “The Temporal Subcutaneous Brow Lift With Orbicularis Oculi Muscle Elastic Flap: Technical Considerations, Systematic Review, and Terminology Standardization,” Facial Plastic Surgery 39, no. 6 (2023): 691–702, 10.1055/a-1953-2304. 36174656

[13] A. Carruthers , J. Carruthers , K. De Boulle , N. Lowe , E. Lee , and M. F. Brin , “Treatment of Crow's Feet Lines and Forehead Lines With Botox (OnabotulinumtoxinA): Development, Insights, and Impact,” Medicine 102, no. S1 (2023): e32496, 10.1097/MD.0000000000032496. 37499083 PMC10374187

[14] J. Carruthers , A. Carruthers , A. Blitzer , N. Eadie , and M. F. Brin , “Treatment of Glabellar Lines With Botox (OnabotulinumtoxinA): Development, Insights, and Impact,” Medicine 102, no. S1 (2023): e32375, 10.1097/MD.0000000000032375. 37499082 PMC10374180

[15] A. Carruthers , J. Carruthers , G. D. Monheit , P. G. Davis , and G. Tardie , “Multicenter, Randomized, Parallel‐Group Study of the Safety and Effectiveness of OnabotulinumtoxinA and Hyaluronic Acid Dermal Fillers (24‐Mg/Ml Smooth, Cohesive Gel) Alone and in Combination for Lower Facial Rejuvenation,” Dermatologic Surgery 36, no. Suppl 4 (2010): 2121–2134, 10.1111/j.1524-4725.2010.01705.x. 21134044

[16] K. R. Coleman and J. Carruthers , “Combination Therapy With BOTOX and Fillers: The New Rejuvnation Paradigm,” Dermatologic Therapy 19, no. 3 (2006): 177–188, 10.1111/j.1529-8019.2006.00072.x. 16784517

[17] S. H. Weinkle , W. P. Werschler , C. F. Teller , et al., “Impact of Comprehensive, Minimally Invasive, Multimodal Aesthetic Treatment on Satisfaction With Facial Appearance: The HARMONY Study,” Aesthetic Surgery Journal 38, no. 5 (2018): 540–556, 10.1093/asj/sjx179. 29244069

[18] H. Sundaram , S. Liew , M. Signorini , et al., “Global Aesthetics Consensus: Hyaluronic Acid Fillers and Botulinum Toxin Type A‐Recommendations for Combined Treatment and Optimizing Outcomes in Diverse Patient Populations,” Plastic and Reconstructive Surgery 137, no. 5 (2016): 1410–1423, 10.1097/PRS.0000000000002119. 27119917 PMC5242215

[19] A. F. Klassen , S. J. Cano , J. C. Grotting , et al., “FACE‐Q Eye Module for Measuring Patient‐Reported Outcomes Following Cosmetic Eye Treatments,” JAMA Facial Plastic Surgery 19, no. 1 (2017): 7–14, 10.1001/jamafacial.2016.1018. 27631534 PMC5247311

[20] L. Donofrio , J. Carruthers , B. Hardas , et al., “Development and Validation of a Photonumeric Scale for Evaluation of Infraorbital Hollows,” Dermatologic Surgery 42, no. Suppl 1 (2016): S251–S258, 10.1097/DSS.0000000000000856. 27661748 PMC5671793

[21] H. A. Gulbitti , S. Waziri , and B. van der Lei , “From Some‐Tox to More‐Tox During the COVID‐19 Pandemic,” Plastic and Reconstructive Surgery 148, no. 4 (2021): 698e–699e, 10.1097/PRS.0000000000008366. PMC8452238 34495914

[22] T. D. Pikoos , S. Buzwell , G. Sharp , and S. L. Rossell , “The Zoom Effect: Exploring the Impact of Video Calling on Appearance Dissatisfaction and Interest in Aesthetic Treatment During the COVID‐19 Pandemic,” Aesthetic Surgery Journal 41, no. 12 (2021): NP2066–NP2075, 10.1093/asj/sjab257. 34146086

[23] B. S. Biesman , J. B. Green , R. George , et al., “A Multicenter, Randomized, Evaluator‐Blinded Study to Examine the Safety and Effectiveness of Hyaluronic Acid Filler in the Correction of Infraorbital Hollows,” Aesthetic Surgery Journal 44, no. 9 (2024): 1001–1013, 10.1093/asj/sjae073. 38573527 PMC11334209

[24] S. Fabi , C. Zoumalan , S. Fagien , S. Yoelin , M. Sartor , and S. Chawla , “A Prospective, Multicenter, Single‐Blind, Randomized, Controlled Study of VYC‐15L, a Hyaluronic Acid Filler, in Adults for Correction of Infraorbital Hollowing,” Aesthetic Surgery Journal 41, no. 11 (2021): NP1675–NP1685, 10.1093/asj/sjab308. 34351386 PMC8520027

[25] J. Woodward , S. E. Cox , K. Kato , F. Urdiales‐Galvez , C. Boyd , and N. Ashourian , “Infraorbital Hollow Rejuvenation: Considerations, Complications, and the Contributions of Midface Volumization,” Aesthetic Surgery Journal Open Forum 5 (2023): ojad016, 10.1093/asjof/ojad016. 36998744 PMC10045888

[26] S. G. Fabi , J. Y. Park , W. W. S. Ho , V. Vachiramon , and S. Dayan , “Aesthetic Considerations for Treating the Asian Patient: Thriving in Diversity International Roundtable Series,” Journal of Cosmetic Dermatology 22, no. 6 (2023): 1805–1813, 10.1111/jocd.15787. 37102267

[27] S. Liew , W. T. L. Wu , H. H. Chan , et al., “Consensus on Changing Trends, Attitudes, and Concepts of Asian Beauty,” Aesthetic Plastic Surgery 44, no. 4 (2020): 1186–1194, 10.1007/s00266-020-01808-w. 32844263

[28] W. T. Wu , S. Liew , H. H. Chan , et al., “Consensus on Current Injectable Treatment Strategies in the Asian Face,” Aesthetic Plastic Surgery 40, no. 2 (2016): 202–214, 10.1007/s00266-016-0608-y. 26893276 PMC4819487

[29] “Juvéderm VOLBELLA With Lidocaine 32G Directions for Use. 73363JR10,” Revision 2020‐07‐10.

[30] “Juvéderm VOLIFT With Lidocaine Diections for Use. 73652JR10,” Revision 2019‐09‐09.

[31] “Juvéderm VOLUMA With Lidocaine Direction for Use. 73650JR10,” Revision 2019‐09‐09.

[32] “Vistabel Summary of Product Characteristics,” AbbVie Ltd. Medicines.ie, accessed February 24, 2025, https://www.medicines.ie/medicines/vistabel‐34207/spc.

[33] S. D'Amato , R. Fragola , P. Bove , et al., “Is the Treatment of the Tear Trough Deformity With Hyaluronic Acid Injections a Safe Procedure? A Systematic Review,” Applied Sciences‐Basel 11, no. 23 (2021): 11489, 10.3390/app112311489.

[34] L. N. Trinh , S. E. Grond , and A. Gupta , “Dermal Fillers for Tear Trough Rejuvenation: A Systematic Review,” Facial Plastic Surgery 38, no. 3 (2022): 228–239, 10.1055/s-0041-1731348. 34192769

[35] F. Urdiales‐Galvez , N. E. Delgado , V. Figueiredo , et al., “Treatment of Soft Tissue Filler Complications: Expert Consensus Recommendations,” Aesthetic Plastic Surgery 42, no. 2 (2018): 498–510, 10.1007/s00266-017-1063-0. 29305643 PMC5840246

[36] C. de la Guardia , A. Virno , M. Musumeci , A. Bernardin , and M. B. Silberberg , “Rheologic and Physicochemical Characteristics of Hyaluronic Acid Fillers: Overview and Relationship to Product Performance,” Facial Plastic Surgery 38, no. 2 (2022): 116–123, 10.1055/s-0041-1741560. 35114708 PMC9188840

